# *Escherichia coli* “TatExpress” strains export several g/L human growth hormone to the periplasm by the Tat pathway

**DOI:** 10.1002/bit.27147

**Published:** 2019-09-02

**Authors:** Isabel Guerrero Montero, Kirsty L. Richards, Chillel Jawara, Douglas F. Browning, Amber R. Peswani, Mickael Labrit, Matthew Allen, Cedric Aubry, Emma Davé, David P. Humphreys, Stephen J. W. Busby, Colin Robinson

**Affiliations:** ^1^ School of Biosciences University of Kent Canterbury United Kingdom; ^2^ School of Biosciences, Institute of Microbiology and Infection University of Birmingham Birmingham United Kingdom; ^3^ Discovery Biology, UCB Celltech Slough United Kingdom

**Keywords:** biopharmaceuticals, *E. coli*, protein secretion, recombinant protein, Tat system

## Abstract

*Escherichia coli* is a heavily used platform for the production of biotherapeutic and other high‐value proteins, and a favored strategy is to export the protein of interest to the periplasm to simplify downstream processing and facilitate disulfide bond formation. The Sec pathway is the standard means of transporting the target protein but it is unable to transport complex or rapidly folding proteins because the Sec system can only transport proteins in an unfolded state. The Tat system also operates to transport proteins to the periplasm, and it has significant potential as an alternative means of recombinant protein production because it transports fully folded proteins. Here, we have tested the Tat system's full potential for the production of biotherapeutics for the first time using fed‐batch fermentation. We expressed human growth hormone (hGH) with a Tat signal peptide in *E. coli* W3110 “TatExpress” strains that contain elevated levels of the Tat apparatus. This construct contained four amino acids from TorA at the hGH N‐terminus as well as the initiation methionine from hGH, which is removed in vivo. We show that the protein is efficiently exported to the periplasm during extended fed‐batch fermentation, to the extent that it is by far the most abundant protein in the periplasm. The protein was shown to be homogeneous, disulfide bonded, and active. The bioassay showed that the yields of purified periplasmic hGH are 5.4 g/L culture whereas an enzyme‐linked immunosorbent assay gave a figure of 2.39 g/L. Separate analysis of a TorA signal peptide linked to hGH construct lacking any additional amino acids likewise showed efficient export to the periplasm, although yields were approximately two‐fold lower.

## Graphical abstract

“TatExpress” is an *E. coli* W3110 strain that contains elevated levels of the Tat apparatus which can be useful for the production of biopharmaceuticals. Here, we have tested the Tat system's full potential for the production of biotherapeutics for the first time using fed‐batch fermentation by expressing human growth hormone (hGH) with a Tat signal peptide in *E. coli* W3110 “TatExpress” strain and wild type.

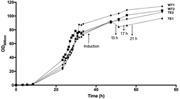

## 1 INTRODUCTION

Many high‐value proteins are produced in *Escherichia coli,* and a favored strategy is to export the protein to the periplasm, usually by the well‐characterized “Sec” pathway (Walsh, 2014). Several strategies have been used, including expression of soluble proteins in the cytoplasm, expression in the form of insoluble inclusion bodies, or export to the periplasm. The latter approach is particularly favored for the production of proteins that contain disulfide bonds, because the periplasm is the only oxidizing compartment in wild‐type (WT) cells (Pooley, Merchante, & Karamata, 1996). Downstream processing is also simplified due to the lowered levels of cytoplasmic contaminants, including DNA (Balasundaram, Harrison, & Bracewell, 2009). Export of proteins to the periplasm is usually achieved by targeting via the well‐characterized Sec pathway, whereby a cleavable Sec‐specific signal peptide is present at the N‐terminus of the target protein. However, the Sec pathway cannot transport some heterologous proteins and attention has focused on the alternative Tat pathway, which transports fully folded proteins by a completely different pathway (reviewed by Natale, Bruser, & Driessen, 2008). Initial studies showed that Tat could export a model protein, green fluorescent protein, at high levels and that the cells were robust under fermentation conditions (Matos et al., 2012). This work was followed by additional studies that showed Tat to be capable of exporting several biotherapeutics, including human growth hormone (hGH), single chain antibody fragments, and interferons (Alanen et al., 2015; Browning et al., 2017; DeLisa, Tullman, & Georgiou, 2003; Matos et al., 2014; Tullman‐Ercek et al., 2007).

hGH is a 22 kDa protein consisting of 191 amino acids (Pooley et al., 1996) used to treat burn injuries, wound healing, hypopituitarism, and obesity (Isaksson, Edén, & Jansson, 1985) because it is safe to use even in high doses (Van Loon, 1998). Because hGH only has two disulfide bonds (Cys53–Cys165 and Cys182–Cys189; Ultsch, Somers, Kossiakoff, & Devos, 1994) and no glycosylation, most of its large‐scale production has used *E. coli* platforms, although a wide range of eukaryotic host systems have been tested (e.g., Ecamilla‐Trevino, Viader‐Salvado, Barrera‐Saldana, & Guerrero‐Olazaran, 2000; Hahm & Chung, 2001). The highest recorded yield is 5 g of recombinant hGH per liter of milk in transgenic cows (Salamone et al., 2006).

A major difficulty in producing hGH in *E. coli* is its aggregation into insoluble bodies that complicate the production process. Until recently, most commercial hGH has been obtained from insoluble bodies that have been solubilized, with the hGH refolded into its active form (Olson et al., 1981; Patra et al., 2000). Recent, novel approaches have managed to produce high quantities of cytosolic hGH in fed‐batch fermentation, with reported yields of up to 678 mg/L protein (Song, Jiang, Wang, & Zhang, 2017).

Although studies exporting hGH to the periplasm have reported relatively low yields (Sockolosky & Szoka, 2013), this is an attractive option due to the ease of downstream processing. In recent studies, we have shown that the Tat system can export hGH with high efficiency and that it is disulfide bonded (Alanen et al., 2015). Our study concluded that although the protein was presumably synthesized and exported in the reduced state, the periplasmic protein was fully disuphide bonded by the normal DSB disulfide bonding system. We have also reported that a new series of engineered *E. coli* strains, which overexpress the Tat system components (termed TatExpress), demonstrate even higher export efficiencies (Browning et al., 2017). These strains bear a modified chromosomal *tatABC* operon, the expression of which is induced by isopropyl‐β‐d‐thiogalactoside (IPTG), so that the Tat system is overexpressed at the same time as the IPTG‐induced target protein. However, the activity of the hGH was not assayed and our studies (Alanen et al., 2015; Browning et al., 2017) used laboratory shake flask systems that bear little resemblance to industrial production processes. Here, we have assessed the feasibility of this system for industrial production using TatExpress *E*. *coli* strains under fed‐batch fermentation conditions. We report that the system is able to produce purified, disulfide bonded, active hGH at levels that are calculated to be between 2 and 5 g/L culture.

## 2 MATERIALS AND METHODS

### 2.1 Bacterial strains and plasmids

Strains and plasmids are summarized in Table 1. *E. coli* strain W3110 was used, together with W3110 TatExpress which was previously described in Browning et al. (2017) alongside the plasmid pKRK7 (pEXT22 expressing TorA–hGH–6His). pKRK38 was derived from pKRK7 to eliminate the TorA signal peptide that precedes the hGH. pKRK38 was made by polymerase chain reaction (PCR) amplification using pKRK7 as a template and primers Mature_hGH_F (5′‐ATGTTCCCAACCATTCCCTTATCCA‐3′) and Mature_hGH_R (5′‐CATACATGTTCCTCTGTGGTAGGGT‐3′) as forward and reverse primers. PCR solutions, enzymes and reaction conditions were as described in Guerrero‐Montero et al. (2019).

**Table 1:** Strains and plasmids used in this work

Strains/plasmids	Description	Source/reference
W3110	*E. coli* K‐12 strain. F−, *λ* ^−^, *IN*(*rrnD‐rrnE)1 rph‐1*	Hayashi et al. (2006)
W3110 TatExpress	W3110 carrying a *ptac* promoter upstream of *tatABCD*	Browning et al. (2017)
pKRK7	pEXT22 expressing TorA–hGH–6His	Browning et al. (2017)
pKRK36	pEXT22 expressing hGH–6His	This work
pCJ14	As pKRK36 but lacking four residues of mature TorA and the hGH initiation methionine	This work

An additional TorA–hGH construct was made removing the four initial amino acids of mature TorA and the N‐terminal methionine of hGH. The construct was made by Gibson assembly in which the hGH–His6 was amplified from plasmid pKRK7 (pEXT22, TorA–hGH–His6) and inserted into a pEXT22 backbone in which the TorA signal sequence had already been cloned. Colonies were screened colony PCR and confirmation of the intended deletion was provided by sequencing from Eurofins Genomics.

**Table:** 

Primer name	Sequence
hGH_del.FOR	AACGCCGCGACGTGCGACTGCGTTCCCAACCATTCCCTTATCCAGGC
hGH_del.REV	TTAATGGTGATGGTGATGGTGGAAGCCACAGCTGCCCTCC
TorA_del.FOR	GTGGAGGGCAGCTGTGGCTTCCACCATCACCATCACCATTAATAAGGATCTATATGACTAG
TorA_del.REV	ATAAGGGAATGGTTGGGAACGCAGTCGCACGTCGCGG

### 2.2 Fed‐batch fermentation

Initial cultures were grown in 50 ml of 2XP media (16 g/L of Bacto‐tryptone/peptone, 10 g/L of yeast extract, 10 g/L NaCl) in 250 ml shake flasks for 6 hr at 37°C, 200 rpm with 1:1,000 antibiotic (5 μl, 1 M kanamycin). One milliliter of culture was transferred to two hundred milliliters of SM6 defined media (Humphreys et al., 1998) and grown aerobically overnight at 30°C, 200 rpm in shake flasks with 1:1,000 antibiotic (5 μl, 1 M kanamycin). On the following day, an equivalent of 300 OD_600_ was used to inoculate fresh defined SM6 media to a final volume of 500 ml in Infors Multifors 1.5 L fermenters (Infors UK Ltd., Reigate, UK). The pH was kept at 7 using 25% (vol/vol) ammonia solution and 25% (vol/vol) sulfuric acid. Dissolved oxygen tension was kept at 30% using gas blending with 100% oxygen where necessary, and the culture was maintained at 30°C until both stirrer and airflow was maximal and then dropped to 25°C. Supplementation of MgSO_4_ occurred when the OD_600_ reached 38–42 (8 ml/L of 1 M MgSO_4_) and of Na_2_HPO_4_ when the OD_600_ reached 54–58 (5 ml/L of 232.8 g/L Na_2_HPO_4_) and when the OD_600_ reached 66–77 (7 ml/L of 232.8 g/L Na_2_HPO_4_). A glycerol feed containing 80% wt/wt glycerol was started at OD_600_ 70, with a continuous feed of 0.01 ml/min. Induction with 9 ml/L of IPTG at a concentration of 4.31 g/L occurred at OD_600_ 75.

### 2.3 Fractionation

Cytoplasm, membrane, and periplasm fractions were prepared by an osmotic‐shock method. Cells were centrifuged at 3,000*g*, 4°C, for 10 min and resuspended in 500 µl of Buffer 1 (100 mM Tris‐acetate pH 8.2, 500 mM sucrose, 5 mM ethylenediaminetetraacetic acid [EDTA] pH 8.0) and 500 µl dH_2_O before addition of 40 µl hen egg white lysozyme (1 mg/ml) and incubation on ice for 5 min. Twenty microliter MgSO_4_ (1 M) was added and the suspension was centrifuged at 20,000*g*, 4°C, 2 min. The supernatant containing the periplasmic fraction was removed and the pellet washed once in 750 µl Buffer 2 (50 mM Tris‐acetate pH 8.2, 250 mM sucrose, 10 mM MgSO_4_). The cell pellet was resuspended in 750 µl Buffer 3 (50 mM Tris‐acetate pH 8.2, 2.5 mM EDTA pH 8.0) and sonicated for 6 × 10 s, amplitude 8 µm to disrupt membranes (Soniprep 150 Plus, Sanyo Gallenkamp; Loughborough, UK). The suspension was centrifuged at 202,000*g*, 4°C, 30 min to sediment the insoluble fraction. Five hundred microliter of the supernatant was removed (designated cytoplasmic fraction). The remainder of the supernatant was discarded and the pellet resuspended in 500 μl Buffer 3 and designated membrane (or insoluble) fraction.

### 2.4 Protein purification

For purification of 6X Histidine‐tagged (C‐term) proteins by Nickel IMAC, 10 ml of the culture post‐induction was taken, centrifuged at 3,000 rpm, 45 min, 4°C. Cell pellet was resuspended in 10 ml/g of chilled Buffer 1 without EDTA and 10 ml/g of milliQ H_2_O and incubated on ice for 30 min before centrifuging at 20,000*g*, 20 min, 4°C (Beckman Avanti J‐ 25, JA 25.5 rotor). Supernatant was taken as the periplasmic fraction and placed into SnakeSkin® dialysis tubing (Thermo Fisher Scientific) and dialysed at 4°C overnight into 50 mM sodium phosphate, 150 mM NaCl, pH 7.2. Using an ÄKTA™ pure protein purification system and a HisTrap HP histidine‐tagged protein column (GE Healthcare, Buckinghamshire, UK) the protein was purified. Storage solution (20% ethanol) was washed off with 10 column volumes (CV) of milliQ H_2_O before adding 5 ml 0.2 M NiCl, followed by another 2 CV milliQ H_2_O wash. Columns were equilibrated with 3 CV equilibration buffer (50 mM sodium phosphate pH 7.2, 0.3 M NaCl) before loading Periplasmic sample and collecting flow through. Unbound matter was removed with 6 CV wash buffer (50 mM sodium phosphate pH 7.2, 50 mM imidazole, 0.3 M sodium chloride) and sample collected as wash. Finally, the 6X Histidine‐tagged protein was eluted with elution buffer with imidazole (50 mM sodium phosphate pH 7.2, 0.3 M NaCl with 250 mM imidazole) and the peaks collected for analysis.

### 2.5 Protein expression analysis by Coomassie and western blot

Protein samples were resolved by reducing sodium dodecyl sulfate‐polyacrylamide gel electrophoresis (SDS‐PAGE) and analyzed using Coomassie blue staining and western blotting. All immunoblotting procedures were as described in Guerrero‐Montero et al. (2019). Periplasmic hGH activity was assayed using an hGH bioassay (PathHunter® Human Growth Hormone Bioassay Kit, Sigma) using the manufacturer's protocol. Standardized OD_10_ periplasmic fractions were diluted 1:500,000 using phosphate‐buffered saline and absorbance was read using a BMG LABTECH SPECTROstar microplate reader at 405 nm, with a reference wavelength at 490 nm. Concentrations were calculated from two independent experiments and all samples were measured in triplicate when calculating periplasmic yield. Periplasmic hGH was also assayed using an hGH ELISA kit (Roche Diagnostics, West Sussex, UK) using the manufacturer's protocol. Concentrations were calculated from two independent experiments and all samples measured in triplicate, and used to calculate average periplasmic yield (in mg/L) by reference to culture OD readings at 600 nm.

### 2.6 Intact protein electrospray liquid chromatography–mass spectrometry (LC‐MS)

The electrospray mass spectrum was recorded on a Bruker micrOTOF‐Q II mass spectrometer. An aliquot of protein in solution, corresponding to approximately 20 pmol of protein, was desalted online by reverse‐phase high performance liquid chromatography (HPLC) on a Phenomenex Jupiter C4 column (5 µm, 300 Ǻ, 2.0 mm × 50 mm) running on an Agilent 1100 HPLC system at a flow rate of 0.2 ml/min using a short water, acetonitrile, 0.05% trifluoroacetic acid gradient. The eluent was monitored at 280 nm and directed into the electrospray source, operating in positive ion mode, at 4.5 kV and mass spectra recorded from 500 to 3,000 m/z. Data were analyzed and deconvoluted to give uncharged protein masses using Bruker's Compass Data Analysis software.

## 3 RESULTS

### 3.1 Fed‐batch fermentation of WT and TatExpress cells expressing TorA–hGH

**Figure 1:**
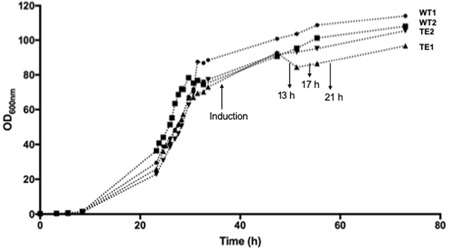
Growth data during fed‐batch fermentation of *Escherichia coli* W3110 WT and TatExpress cells expressing TorA–hGH. Duplicate cultures were analyzed of WT cells (WT1, WT2) and TatExpress (TE1, TE2), with OD_600 nm_ values shown. Fermentation was carried out at 30°C as detailed in Materials and Methods. At the indicated times, the cultures were induced with IPTG (0.1 mM) and samples were removed for analysis. IPTG, isopropyl‐β‐d‐thiogalactoside; TorA–hGH, TorA signal peptide linked to hGH; WT, wild‐type

We first expressed a construct comprising the TorA signal peptide linked to hGH (TorA–hGH) in W3110 *E. coli* TatExpress cells under fed‐batch fermentation conditions as detailed in Materials and Methods. The construct was previously used in shake flask studies (Browning et al., 2017) and comprises the signal peptide of *E. coli* TMAO reductase (TorA) linked to hGH via a four‐residue linker (the first four residues of mature TorA; AQAA). The construct encodes the initiation methione of hGH (which is removed in vivo) and also contains a C‐terminal 6‐His tag. Parallel cultures were carried out with the construct expressed in WT W3110 cells. Figure 1 shows the growth curves from the cultures; duplicate WT and TatExpress cultures reached ODs of approximately 100 and Figure 1 shows the point at which induction of TorA–hGH and TatABC synthesis (in TatExpress) was induced using IPTG.

**Figure 2:**
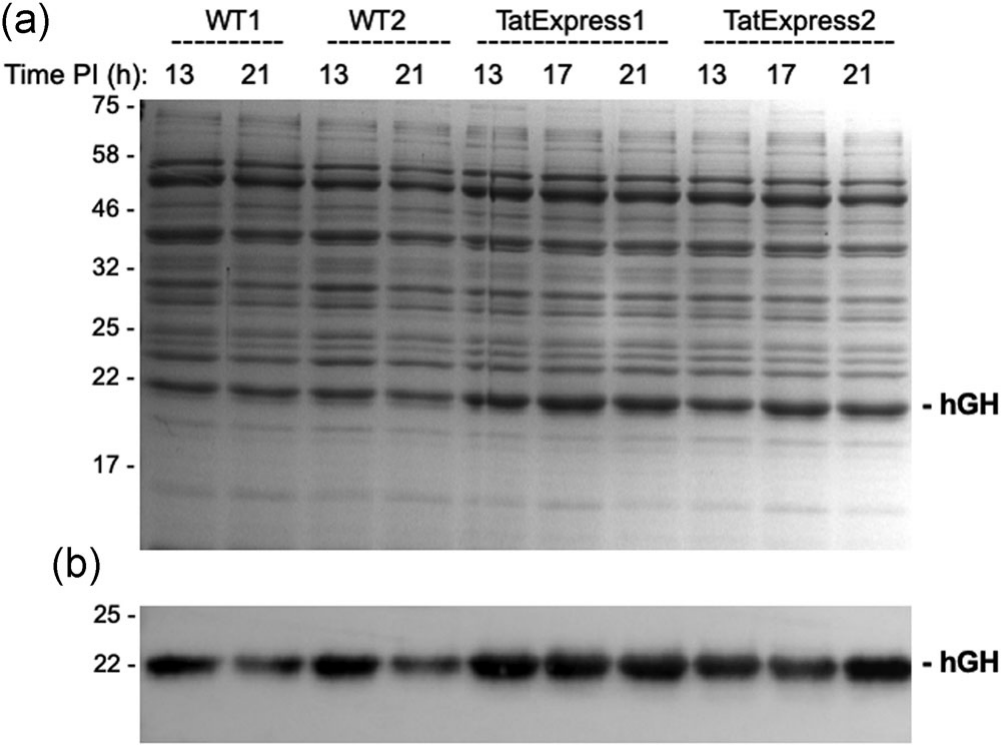
Tat‐dependent export of TorA–hGH in W3110 wild‐type (WT) and TatExpress W3110 cells. Samples from duplicate fed‐batch fermentation cultures of WT and TatExpress expressing TorA–hGH were removed at the indicated times postinduction and fractionated to yield periplasmic samples. The periplasmic samples were analyzed by Coomassie blue stained gels (a) and immunoblotting using antibodies to the His tag on hGH (b). For each lane a normalized amount of protein has been loaded, equivalent to OD_600_ 0.08 AU total cells (Coomassie blue stained gels), and OD_600_ 0.008 AU (immunoblots). Mobilites of molecular weight markers (in kDa) are shown on the left. TorA–hGH, TorA signal peptide linked to hGH

Samples were removed 13, 17, and 21 hr after induction and the cells were fractionated to generate cytoplasmic, membrane and periplasmic samples. The periplasmic samples were analyzed by immunoblotting to detect the hGH and the same samples were analyzed using Coomassie‐stained SDS‐PAGE gels to analyse the proteome of the periplasmic samples. Figure 2 shows the 13 and 21 hr samples from the two WT cultures, which indicate the presence of a clear hGH protein band in the periplasm. The Coomassie‐stained gel shows the presence of a 22 kDa protein, the relative abundance of which matches the strength of the blot signals. This was confirmed to be hGH (see below). Also analyzed were 13, 17, and 21 hr samples from the two TatExpress cultures, and the data show that the hGH blot signals are significantly higher than those of the WT cultures, confirming that TatExpress cells export this construct with much higher efficiency as observed by Browning et al. (2017) in shake flask culture. The abundance of the 22 kDa protein is correspondingly greater in the Coomassie‐stained gels, providing further evidence that this is indeed hGH.

**Figure 3:**
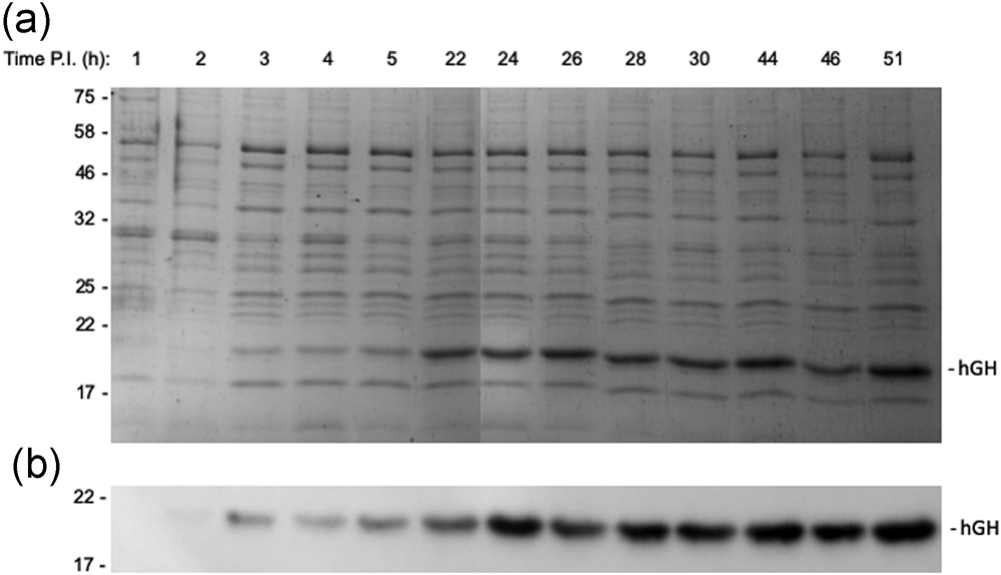
High‐level export of TorA–hGH during extended fed‐batch fermentation of TatExpress cells. TorA–hGH was expressed in TatExpress cells in a fed‐batch fermentation system and samples were removed at the indicated times postinduction (PI). The samples were fractionated to generate periplasm samples which were then analyzed on Coomassie blue stained gels (a) and by immunoblotting using antibodies to the His tag on hGH (b). hGH, human growth hormone; TorA–hGH, TorA signal peptide linked to hGH

Figure 3 shows a time course analysis of the abundance of the periplasmic hGH after induction of synthesis in TatExpress cells. Samples were taken from 2 to 51 hr after induction and the blot shows a steady increase in hGH level over this time period. Equal numbers of cells were used in each sample, so this reflects an increase in the amount of hGH per cell. The Coomassie gel confirms that the abundant 22 kDa protein is indeed hGH, because its abundance increases in parallel and the protein is virtually absent in the induction time point samples. Analysis of the later time points demonstrates that hGH is by far the most abundant periplasmic protein, which shows that Tat is exporting high amounts of protein.

**Figure 4:**
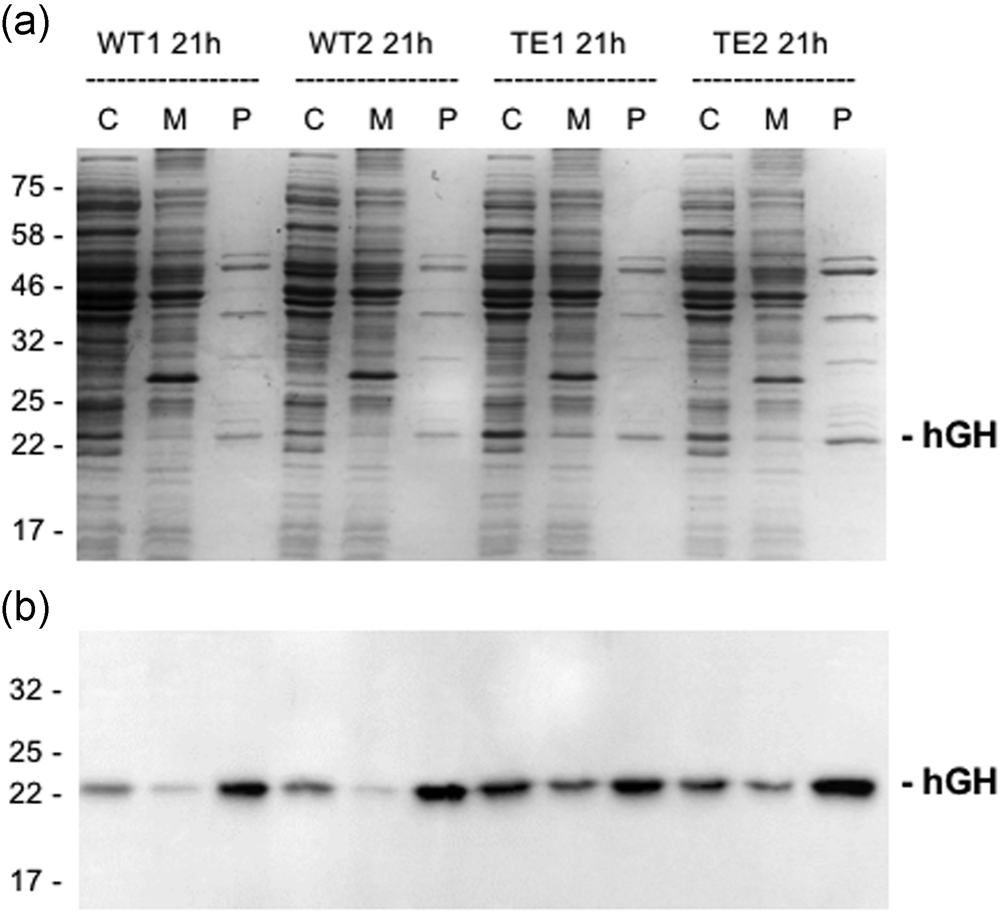
Fractionation of samples from fed‐batch fermentation of W3110 wild‐type (WT) and TatExpress cells expressing TorA–hGH. Samples from the 21 hr time point of fed‐batch fermentation cultures of WT and TatExpress (TE) expressing TorA–hGH (shown in Figure 2) were fractionated to yield cytoplasm, membrane, and periplasm samples (C, M, and P). The samples were analyzed by Coomassie blue stained gels (a) and immunoblotting using antibodies to the His tag on hGH (b). For each lane a normalized amount of protein has been loaded, equivalent to OD_600_ 0.08 AU total cells (Coomassie blue stained gels), and OD_600_ 0.008 AU (immunoblots). Mobilites of molecular weight markers (in kDa) are shown on the left. TorA–hGH, TorA signal peptide linked to hGH

**Figure 5:**
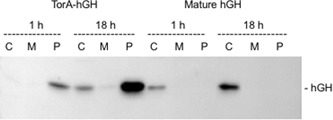
Mature hGH is not exported during fed‐batch fermentation of TatExpress. Mature‐size hGH and TorA–hGH were expressed in TatExpress cells using fed‐batch fermentation systems. Samples were removed 1 and 18 hr postinduction and fractionated to generate cytoplasm, membrane, and periplasm samples (C, M, and P) which were then analyzed by immunoblotting using antibodies to the His tag on hGH. hGH, human growth hormone; TorA–hGH, TorA signal peptide linked to hGH

We have carried out controls for fractionation artifacts in previous studies, and the periplasmic proteome is clearly distinct from that of the cytoplasm, suggesting that contamination of cytoplasmic proteins is minimal (e.g., Matos et al., 2012). However, to confirm this point we fractionated samples at the 21 hr time point and the data are shown in Figure 4. The results show that the fractions are indeed “clean” with the periplasmic proteome clearly distinct from those of the cytoplasm and membrane fractions. To further confirm that hGH does not reach the periplasmic fraction “spontaneously” we expressed hGH lacking any form of signal peptide. Mature hGH was expressed in parallel with TorA–hGH and samples were fractionated after 1 and 18 hr induction (Figure 5). The immunoblot shows that the bulk of hGH is in the periplasm in the TorA–hGH culture, as expected, with a minimal level of protein present in the cytoplasm at the 18 hr point. In contrast, hGH is found exclusively in the cytoplasm in the sample expressing mature hGH, with none detected in the periplasmic fraction. These data confirm that the presence of hGH in the periplasm of the TorA–hGH cultures is due to Tat‐dependent export and not contamination by cytoplasmic fraction or spontaneous transfer across the plasma membrane.

It is notable that the total level of hGH in the culture producing mature hGH is approximately sixfold lower than that in the TorA–hGH culture. This reflects a phenomenon observed previously in our shake flask studies that hGH is subjected to rapid turnover in the cytoplasm (Alanen et al., 2015). Clearly, this protein is more stable in the periplasm, and this is another advantage of using an effective protein export strategy for the production of this particular protein.

### 3.2 TAT‐exported hGH is homogeneous and cleaved at the correct signal peptidase site

**Figure 6:**
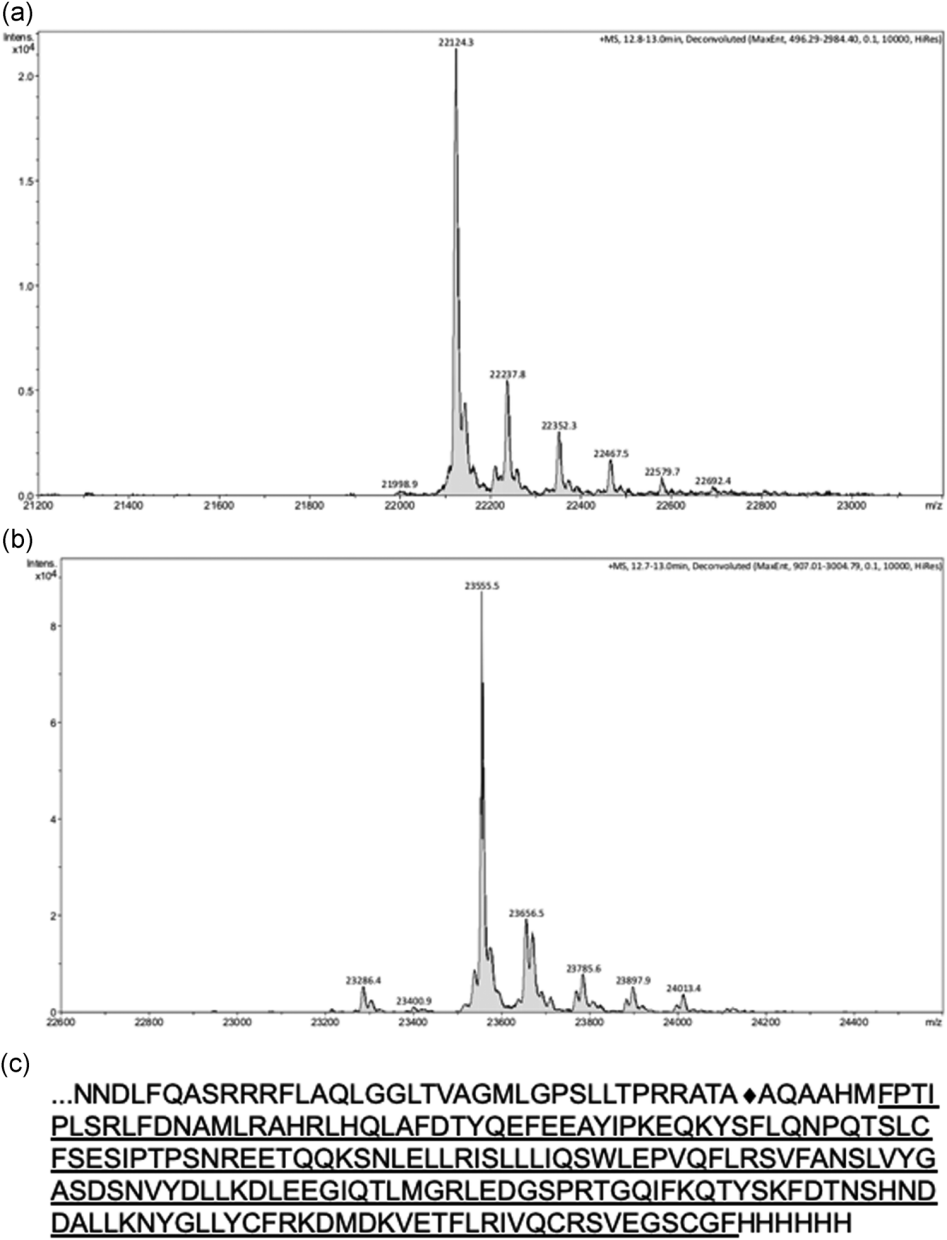
Exported periplasmic hGH is homogeneous and cleaved at the correct site. Periplasmic hGH was purified by affinity chromatography (see Figure S1) and subjected to mass spectrometry analysis as detailed in Materials and Methods. Commercial hGH was analyzed in an identical manner. The mass spectra for commercial hGH is shown in panel (a) and for periplasmic hGH in (b). (c) Amino acid sequence of TorA–hGH–H6. The C‐terminal half of the TorA signal peptide is shown, along with the first five amino acids of the mature TMAO protein and the initial methionine for the hGH protein. The signal peptidase cleavage site between ATA and AQA is denoted by ♦. The sequence for the commercial hGH is underlined, and this is followed by the 6‐histidine tag. hGH, human growth hormone

To assess the homogeneity of the exported hGH, we performed mass spectrometry analysis of the purified protein, using purified commercial hGH as a standard (provided with the Pathfinder Bioassay—see below). The exported, periplasmic hGH construct used in this study should contain additional amino acids when compared to commercial hGH, specifically the four amino acid linker from the mature TorA protein (TMAO reductase) at the N‐terminus, the initiation methionine (cleaved from hGH in vivo) and the 6‐His tag at the C‐terminus (Figure 6). The combined molecular weight of the additional amino acids is 609.7 for the N‐terminal residues and 822.86 for the C‐terminal His tag (1,432.56 in total). The predicted molecular weights are 22,129.05 for the commercial hGH, and 23,561.5 for the exported hGH. As shown in Figure 6, mass spectrometry analysis of commercial hGH gives a single prominent peak corresponding to 22,124.3 Da, which matches almost exactly with the predicted molecular mass, having only a difference of 4.75 Da, four of which are a consequence of the formation of two disulfide bonds. We purified the Tat‐exported periplasmic hGH using affinity chromatography and an analysis of this protein again shows a single prominent peak, and the mass of 23,555 Da is again very close to, but 6 Da smaller than the predicted mass of 23,561 Da. The presence of two disulfide bonds again accounts for the difference. In both cases, the 1–2 Da deviation from the predicted protein masses is within the error range for the mass spectrometer.

Other smaller peaks are observed in both samples, which appear to be an artifact of the hGH mass spectrometry because they are identical in the two samples. This implies that the hGH produced in this study is (a) as homogeneous as the commercial protein; (b) processed at the correct site; and (c) fully disulfide bonded; there is no indication of a peak corresponding to the reduced form.

**Figure 7:**
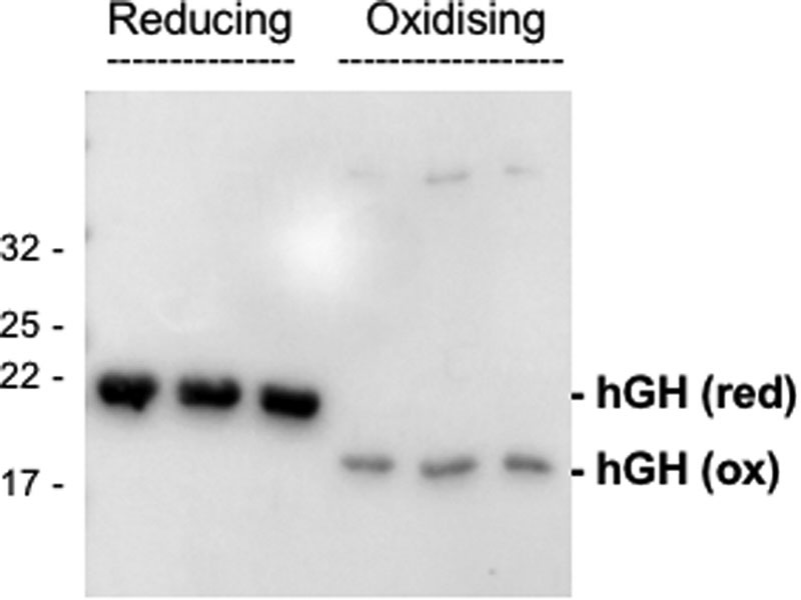
Periplasmic hGH is disulfide bonded after fed‐batch fermentation. Samples of the periplasmic fraction from the 51 hr time point shown in Figure 3 were run (in triplicate) on an SDS polyacrylamide gel under standard reducing conditions (“reducing” or in the absence of reducing agent (“oxidizing”). The samples were immunoblotted using antibodies to the C‐terminal His tag on hGH. hGH “ox” and “red”: Oxidized and reduced forms of hGH, respectively. Mobilties of molecular mass markers (in kDa) are shown on the left. hGH, human growth hormone; SDS, sodium dodecyl sulfate

To confirm that the hGH is indeed disulfide bonded, we ran samples of the periplasmic samples from the 51 hr time point shown in Figure 3 on an SDS‐PAGE gel in the presence and absence of reducing agent. The oxidized, disulfide bonded form of hGH was shown to migrate more rapidly than the reduced form (Alanen et al., 2015) and Figure 7 shows the periplasmic hGH from the fed‐batch fermentation likewise runs more rapidly under oxidizing conditions, further confirming that the protein is disulfide bonded. It is unclear why the intensity of the blot signal is lower; presumably, the C‐terminal His tag is less exposed in the oxidized protein and the antibodies bind less effectively.

### 3.3 High yields of active protein are obtained from fed‐batch fermentation using TorA–hGH

To quantify the yield of periplasmic hGH and to assess its activity, we used a commercially available hGH bioassay (PathHunter® Human Growth Hormone Bioassay Kit, Sigma). This uses engineered cells in which one fragment of β‐galactosidase is present on the hGH receptor and the complementary fragment is present on a phosphor‐tyrosine SH2‐domain‐containing protein that is only able to bind the hGH receptor once it is activated. Binding of hGH to its receptor results in receptor phosphorylation by a cytosolic tyrosine kinase such as JAK1, enabling the SH2‐EA fusion protein to bind the phosphorylated receptor and generate active β‐galactosidase. Enzymatic activity is quantitatively measured using a chemiluminescent substrate, and the expected result is a dose‐response sigmoidal function where the phosphorylation of the receptor will be dependent on the amount of hGH in the sample. A standard curve was produced with the supplied commercial hGH and all samples were done in triplicate and with dilution factors in the range of the standard curve (see Figure S2). This assay gave a figure of 5.4 g hGH per litre of fed‐batch culture for the purified periplasmic hGH shown in Figure S1.

We also calculated hGH levels using a commercial enzyme‐linked immunosorbent assay (ELISA), which uses prebound antibodies to hGH on the surface of the microplate modules. The sample to analyse was added in triplicate (with various dilution factors) to the wells, and the hGH present in the samples binds to the anti‐hGH antibodies. Afterwards a digoxigenin‐labeled antibody to hGH is added which binds to the hGH as well. An antibody to digoxigenin conjugated to peroxidase is added and binds to the digoxigenin, and finally the peroxidase substrate ABTS is added. The peroxidase catalyzes the cleavage of the substrate yielding a colored reaction product that can be measured using a plate reader that reads at 405 and 490 nm. The absorbance is directly correlated to the level of hGH present in the sample and can be determined by comparison to the calibration curve (shown in Figure S3). Using this ELISA the concentration of the purified periplasmic hGH was calculated to be 2.39 g/L culture. This is lower than the figure obtained using the bioassay, but because the assays are so different it is difficult to determine which is more accurate.

### 3.4 Export of TorA–hGH lacking the four‐residue linker between the signal peptide and hGH

**Figure 8:**
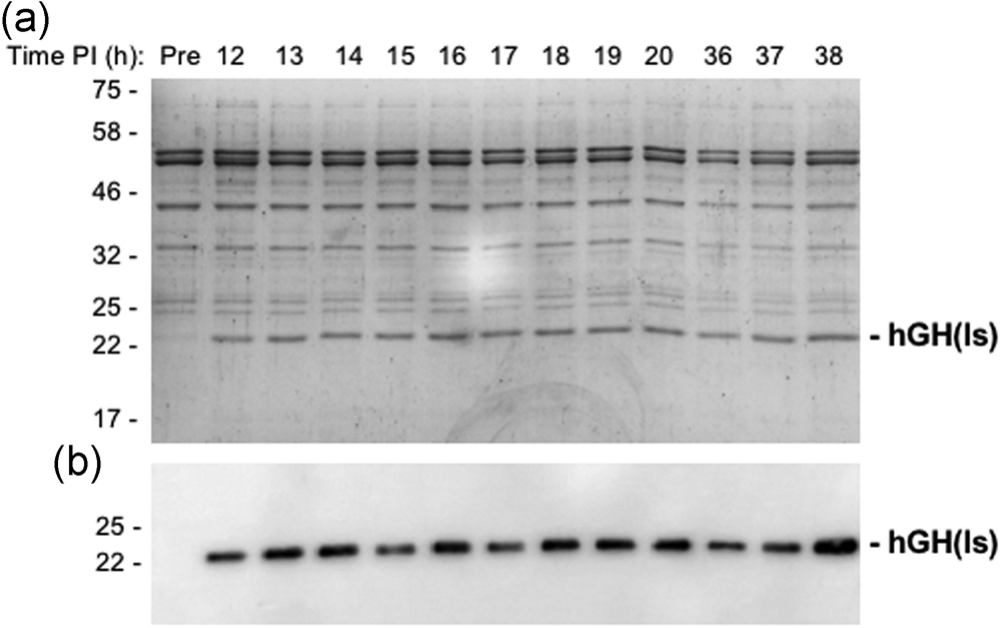
Export of “linker‐less” TorA–hGH (hGH(ls)) during fed‐batch fermentation of TatExpress cells. TorA–hGH lacking a four‐residue linker and the hGH initiation methionine was expressed in TatExpress cells in a fed‐batch fermentation system and samples were removed at the indicated times postinduction (PI). The samples were fractionated to generate periplasm samples which were then analyzed on Coomassie blue stained gels (a) and by immunoblotting using antibodies to the His tag on hGH (b)

The above experiments were all carried out using a construct that comprises the TorA signal peptide, four residues from the N‐terminus of mature TorA and hGH. This construct was used to compare results with shake flask studies carried out using the same construct (Browning et al., 2017), but we considered it important to assess the export of a “linker‐less” construct lacking any linker residues, because industrial processes are unlikely to incorporate such linkers. We therefore removed the four residues and also removed the hGH initiation methione so that the processed form would correspond to authentic hGH. We carried out fed‐batch fermentation studies on this “clean” construct and the periplasmic fractions are analyzed in Figure 8. The immunoblot and gel data show that hGH production is induced and that high levels of hGH are exported to the periplasm over the 38 hr induction period. As with the original construct, periplasmic hGH accumulates to the extent that it is a highly abundant periplasmic protein. The data thus show that removal of the four‐residue linker does not block export of hGH, and that export proceeds in the absence of the hGH initiation methionine.

**Figure 9:**
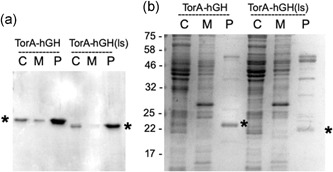
Comparison of export rates for hGH containing or lacking four additional residues at the N‐terminus. TorA–hGH constructs containing four additional residues (as in Figures 2 and 3) or “linker‐less”, lacking any additional residues between the signal peptide and mature protein (as in Figure 8; denoted hGH(ls)) were expressed in TatExpress cells as in the above Figures. After an induction period of 40 hr, cells were fractionated into cytoplasm, membrane, and periplasm samples (C, M, and P) which were analyzed by immunoblotting (a) and on Coomassie‐stained SDS gels (b). * denote the mobilities of the mature hGH proteins. TorA–hGH, TorA signal peptide linked to hGH

Figure 9 shows a direct comparison of the export of the two hGH forms, in which fermentation samples were fractionated into cytoplasm, membrane, and periplasm extracts, which were then blotted for the presence of hGH. The data show that in each case, the majority of hGH is present in the periplasm, confirming that both constructs are efficiently exported. However, the hGH immunoblot signal after export of the linker‐less construct is less intense than that of the original construct containing the additional four residues (Figure 9a). In addition, analysis of the stained gel in Figure 9b shows that while the linker‐less periplasmic hGH is a major band, the abundance is not as striking as that of the periplasmic accumulation of hGH observed in Figures 2 and 3. We, therefore, believe that the linker‐less hGH is exported with lowered efficiency, and quantification of the blots suggests that, on average, its export is in the region of 50% as efficient as that of the original TorA–hGH construct.

## 4 DISCUSSION

The Tat system has been proposed to offer a viable alternative to the Sec pathway for the export of high‐value proteins to the bacterial periplasm, but its potential for the production of biotherapeutic proteins has not been fully explored in previous studies. This is primarily because the majority of those studies were carried out using laboratory shake flask culture systems, whereas industrial processes almost invariably use fed‐batch fermentation. Here, we have used fed‐batch fermentation systems to test the robustness of the TatExpress cells and the yields of hGH that are obtained after export to the periplasm.

A major aim in this work was to test for any deleterious effects due to the increased expression of the TatABC membrane proteins in the TatExpress strain. While the TatABC proteins are expressed to a much lower extent than TorA–hGH, the increased expression of any membrane protein can potentially lead to cell stress and lowered productivity in extended fed‐batch fermentation systems. Here, we directly compared the growth characteristics of the TatExpress strain with its parental W3110 strain and we observed no significant differences. Clearly, TatExpress strains are viable production hosts.

In terms of target protein yield, we consistently observe that the cells export TorA–hGH throughout an extended induction period and it is notable that the exported hGH is by far the most abundant periplasmic protein by the end of the induction period. In control tests, mature‐size hGH is not exported at all and we can conclude that TorA–hGH is exported by the TorA signal peptide as reported in shake flask studies (Alanen et al., 2015). The yields of protein are high: the bioassay indicates a yield of over 5 g/L active purified protein while the ELISA gives a figure of 2.39 g/L. This is one of the highest yields reported and clear evidence that this platform has potential for industrial use. Nevertheless, further study would serve to illustrate the potential in more detail, and it is notable that, while a “clean” fusion of a Tat signal peptide and hGH is efficiently exported, the presence of a short linker region appears to enhance export. An optimal signal peptide‐passenger protein junction may be an important factor for efficient Tat‐dependent export, as previously suggested by Tullman‐Ercek et al. (2007).

In summary, we have shown that a model biotherapeutic protein can be exported by the Tat system in high amounts, and that it is homogeneous, disulfide bonded and active. It will be of interest to conduct further studies to assess the full capability of the Tat system, and in particular to explore its potential for the export of more complex proteins.

## CONFLICT OF INTERESTS

The authors declare that there are no conflict of interests.

## Supporting information

Supporting information

## References

[bit27147-bib-0001] Alanen, H. I. , Walker, K. L. , Lourdes Velez Suberbie, M. , Matos, C. F. R. O. , Bönisch, S. , Freedman, R. B. , … Robinson, C. (2015). Efficient export of human growth hormone, interferon α2b and antibody fragments to the periplasm by the *Escherichia coli* Tat pathway in the absence of prior disulfide bond formation. Biochimica et Biophysica Acta, 1853(3), 756–763. 25554517 10.1016/j.bbamcr.2014.12.027

[bit27147-bib-0002] Balasundaram, B. , Harrison, S. , & Bracewell, D. G. (2009). Advances in product release strategies and impact on bioprocess design. Trends in Biotechnology, 27(8), 477–485. 19573944 10.1016/j.tibtech.2009.04.004

[bit27147-bib-0003] Browning, D. F. , Richards, K. L. , Peswani, A. R. , Roobol, J. , Busby, S. J. W. , & Robinson, C. (2017). *Escherichia coli* “TatExpress” strains super‐secrete human growth hormone into the bacterial periplasm by the Tat pathway. Biotechnology and Bioengineering, 114, 2828–2836. 28842980 10.1002/bit.26434 PMC5698719

[bit27147-bib-0004] DeLisa, M. P. , Tullman, D. , & Georgiou, G. (2003). Folding quality control in the export of proteins by the bacterial twin‐arginine translocation pathway. Proceedings of the National Academy of Sciences of the United States of America, 100(10), 6115–6120. 12721369 10.1073/pnas.0937838100 PMC156335

[bit27147-bib-0005] Ecamilla‐Treviño, L. L. , Viader‐Salvadó, J. M. , Barrera‐Saldaña, H. A. , & Guerrero‐Olazarán, M. (2000). Biosynthesis and secretion of recombinant human growth hormone in *Pichia pastoris* . Biotechnology Letters, 22, 109–114.

[bit27147-bib-0006] Guerrero Montero, I. , Dolata, K. M. , Schlüter, R. , Malherbe, G. , Sievers, S. , Zühlke, D. , … Robinson, C. (2019). Comparative proteome analysis in an *Escherichia coli* CyDisCo strain identifies stress responses related to protein production, oxidative stress and accumulation of misfolded protein. Microbial Cell Factories, 18, 19. 30696436 10.1186/s12934-019-1071-7 PMC6350376

[bit27147-bib-0007] Hahm, M. S. , & Chung, B. H. (2001). Secretory expression of human growth hormone in *Saccharomyces cerevisiae* using three different leader sequences. Biotechnology and Bioprocess Engineering, 6, 306–309.

[bit27147-bib-0008] Hayashi, K. , Morooka, N. , Yamamoto, Y. , Fujita, K. , Isono, K. , Choi, S. , … Horiuchi, T. (2006). Highly accurate genome sequences of *Escherichia coli* K‐12 strains MG1655 and W3110. Molecular Systems Biology, 2(1), 2006.0007. 10.1038/msb4100049 PMC1681481 16738553

[bit27147-bib-0009] Humphreys, D. P. , Vetterlein, O. M. , Chapman, A. P. , King, D. J. , Antoniw, P. , Suitters, A. J. , … Stephens, P. E. (1998). F(ab′)2 molecules made from *Escherichia coli* produced Fab′ with hinge sequences conferring increased serum survival in an animal model. Journal of Immunological Methods, 217, 1–10. 9776570 10.1016/s0022-1759(98)00061-1

[bit27147-bib-0010] Isaksson, O. G. P. , Eden, S. , & Jansson, J. (1985). Mode of action of pituitary growth hormone on target cells. Annual Review of Physiology, 47, 483–499. 10.1146/annurev.ph.47.030185.002411 3888078

[bit27147-bib-0011] Matos, C. F. R. O. , Branston, S. D. , Albiniak, A. , Dhanoya, A. , Freedman, R. B. , Keshavarz‐Moore, E. , … Robinson, C. (2012). High‐yield export of a native heterologous protein to the periplasm by the tat translocation pathway in *Escherichia coli* . Biotechnology and Bioengineering, 109(10), 2533–2542. 22539025 10.1002/bit.24535

[bit27147-bib-0012] Matos, C. F. R. O. , Robinson, C. , Alanen, H. I. , Prus, P. , Uchida, Y. , Ruddock, L. W. , … Keshavarz‐Moore, E. (2014). Efficient export of prefolded, disulfide‐bonded recombinant proteins to the periplasm by the Tat pathway in *Escherichia coli* CyDisCo strains. Biotechnology Progress, 30(2), 281–290. 24376243 10.1002/btpr.1858

[bit27147-bib-0013] Natale, P. , Brüser, T. , & Driessen, A. J. M. (2008). Sec‐ and Tat‐mediated protein secretion across the bacterial cytoplasmic membrane‐‐distinct translocases and mechanisms. Biochimica et Biophysica Acta, 1778(9), 1735–1756. 17935691 10.1016/j.bbamem.2007.07.015

[bit27147-bib-0014] Olson, K. C. , Fenno, J. , Lin, N. , Harkins, R. N. , Snider, C. , Kohr, W. H. , … Stebbing, N. (1981). Purified human growth hormone from *E. coli* is biologically active. Nature, 293, 408–411. 7024824 10.1038/293408a0

[bit27147-bib-0015] Patra, A. K. , Mukhopadhyay, R. , Mukhija, R. , Krishnan, A. , Garg, L. C. , & Panda, A. K. (2000). Optimization of inclusion body solubilization and renaturation of recombinant human growth hormone from *Escherichia coli* . Protein Expression and Purification, 18, 182–192. 10686149 10.1006/prep.1999.1179

[bit27147-bib-0016] Pooley, H. M. , Merchante, R. , & Karamata, D. (1996). Overall protein content and induced enzyme components of the periplasm of *Bacillus subtilis* . Microbial Drug Resistance, 2(1), 9–15. 9158717 10.1089/mdr.1996.2.9

[bit27147-bib-0017] Salamone, D. , Barañao, L. , Santos, C. , Bussmann, L. , Artuso, J. , Werning, C. , … Melo, C. (2006). High level expression of bioactive recombinant human growth hormone in the milk of a cloned transgenic cow. Journal of Biotechnology, 124, 469–472. 16716426 10.1016/j.jbiotec.2006.01.005

[bit27147-bib-0018] Sambrook, J. , & Russell, D. W. (2001). Molecular cloning: A laboratory manual. Cold Spring Harbor, NY: Cold Spring Harbor Laboratory Press.

[bit27147-bib-0019] Sockolosky, J. T. , & Szoka, F. C. (2013). Periplasmic production via the pET expression system of soluble, bioactive human growth hormone. Protein Expression and Purification, 87(2), 129–135. 23168094 10.1016/j.pep.2012.11.002 PMC3537859

[bit27147-bib-0020] Song, H. , Jiang, J. , Wang, X. , & Zhang, J. (2017). High purity recombinant human growth hormone (rhGH) expression in *Escherichia coli* under *phoA* promoter. Bioengineered, 8(2), 147–153. 27459425 10.1080/21655979.2016.1212137 PMC5398570

[bit27147-bib-0021] Tullman‐Ercek, D. , DeLisa, M. P. , Kawarasaki, Y. , Iranpour, P. , Ribnicky, B. , Palmer, T. , … Georgiou, G. (2007). Export pathway selectivity of *Escherichia coli* twin arginine translocation signal peptides. Journal of Biological Chemistry, 282(11), 8309–8316. 17218314 10.1074/jbc.M610507200 PMC2730154

[bit27147-bib-0022] Ultsch, M. H. , Somers, W. , Kossiakoff, A. A. , & Devos, A. M. (1994). The crystal structure of affinity‐matured human growth hormone at 2 Å resolution. Journal of Molecular Biology, 236, 286–299. 8107110 10.1006/jmbi.1994.1135

[bit27147-bib-0023] Van Loon, K. (1998). Safety of high doses of recombinant human growth hormone. Hormone Research, 49, 78–81. 9716832

[bit27147-bib-0024] Walsh, G. (2014). Biopharmaceutical benchmarks 2014. Nature Biotechnology, 32, 992–1000. 10.1038/nbt.3040 25299917

