# Supplementary material for: Escherichia coli “TatExpress” strains export several g/L human growth hormone to the periplasm by the Tat pathway
Source: Biotechnol Bioeng. 2019 Sep 2;116(12):3282–91. doi: 10.1002/bit.27147 (PMC6907408; doi:10.1002/bit.27147)
Supplement: Supplementary file 1 — Supporting information [file BIT-116-3282-s001.pdf]

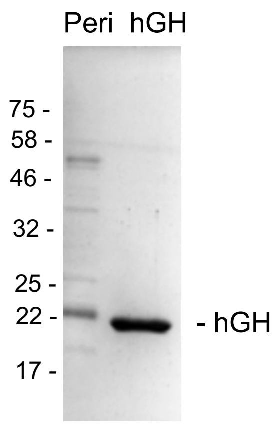

Supplementary Figure 1. Purification of periplasmic hGH after export during fed-batch fermentation. The periplasmic fraction generated from 10 ml of a fed-batch fermentatio0n culture of TatExpress expressing TorA-hGH as in Figures 2 and 3. hGH was purified by IMAC chromatography and the Figure shows a Coomassie-stained gel of the purified hGH (hGH) as well as the periplasm sample used as starting material (peri).

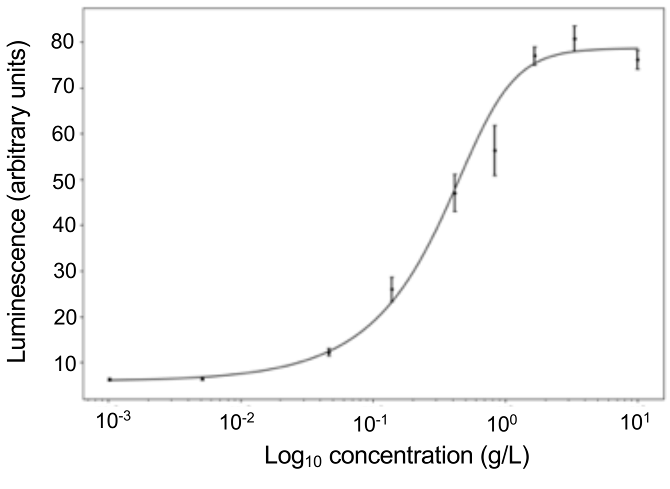

Supplementary Figure 2. Quantification of hGH concentration using the PathFinder bioassay. An hGH concentration standard curve was constructed using the supplied commercial hGH and all samples were analysed in triplicate using dilution factors in the range of the standard curve. The standard curve was adjusted using numpy, matplotlib.pyplot and scipy in Python.

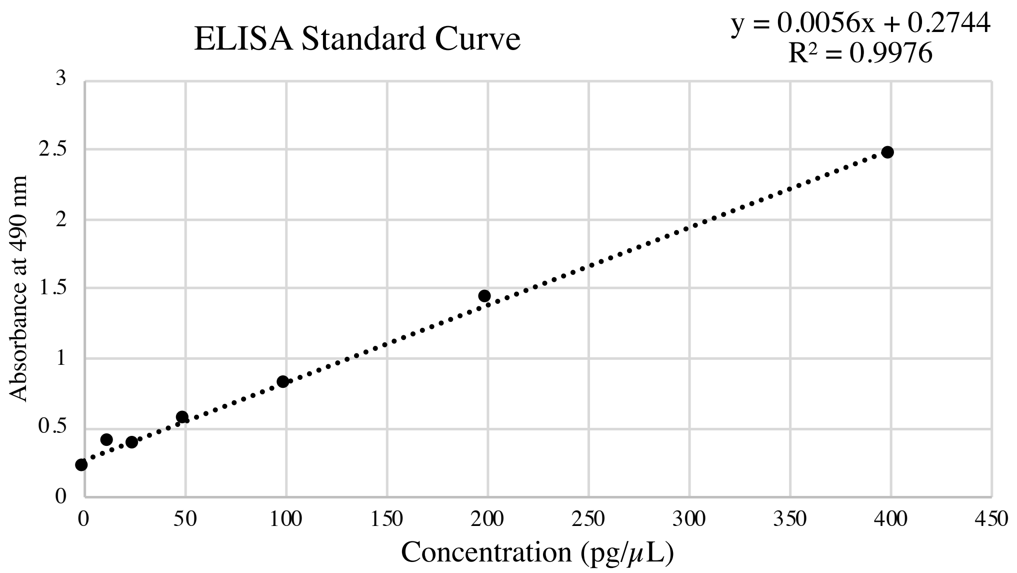

Supplementary Figure 3: Calibration curve and quantification of fed-batch fermentation samples obtained using an hGH ELISA kit. The calibration curve obtained from the ELISA kit using the standard hGH provided is shown, with concentration shown on the X axis (picogram per µL) and Absorbance at 490 nm shown on the Y axis. The purified periplasmic hGH was found to be present at a concentration that corresponds to 2.39 g hGH per litre of culture.

Supplementary material 4. Literature figures for the production of hGH in different host cells

E. coli periplasm (Sec-dependent export)	1.4 mg/L	Sockolosky & Szoka 2013
E. coli cytoplasm	623 mg/L	Song et al., 2017
E. coli cytoplasm	511 mg/L	Levarski et al., 2014
Pichia pastoris secretion	49 mg/L	Ecamilla-Trevino et al., 2000
Pichia pastoris secretion	160 mg/L	Orman et al., 2009
E. coli cytoplasm	150 mg/L	Murad et al., 2014
Goat milk	60 mg/L	Archer et al., 1995
Saccharomyces cerevisiae	0.9 mg/L	Hahm and Chung
E. coli periplasm (Sec-dependent export)	15-25 µg/ml/1 A550 unit	Chang et al., 1987

References:

Sockolosky, J. T., & Szoka, F. C. (2013). Periplasmic production via the pET expression system of soluble, bioactive human growth hormone. Protein Expression and Purification, 87, 129–135.

Song, H, Jiang, J., Wang, Wang, X. and Zhang, J. (2017). High purity recombinant human growth hormone (rhGH) expression in Escherichia coli under phoA promoter. Bioengineered 8, 147-154.

Levarski, Z., Šoltýsová, A., Krahulec, J., Stuchlík S., Turňa, J. (2014). High-level expression and purification of recombinant human growth hormone produced in soluble form in *Escherichia coli*. Protein Exp Purification 100:40-7;

Ecamilla-Trevino, L.L., Viader-Salvado, J.M., Barrera-Saldana, H.A, Guerrero- Olazaran, M. (2000). Biosynthesis and secretion of recombinant human growth hormone in Pichia pastoris, Biotechnol. Lett. 22, 109–114.

Orman, M.A., Calik, P., Ozdamar, T.H. (2009). The influence of carbon sources on recombinant-human- growth-hormone production by Pichia pastoris is dependent on phenotype: a comparison of Muts and Mut+ strains. Biotechnol. Appl. Biochem. 52, 245-255.

Murad, H., Ali, B., Makeya, R., Abbada, A.Q. (2014). Gene 542, 69-76. Prokaryotic overexpression of TEV-rhGH and characterization of its polyclonal antibody.

Archer, J.S., Kennan, W.S., gould, M.N., Bremal, R.D. (1994). Human growth hormone (hGH) secretion in milk of goats after direct transfer of the hGH gene into the mammary gland by using replication-defective retrovirus vectors. Proc. Natl. Acad. Sci USA 91, 6840-6844.

Hahm, M.S., Chung, B.H. (2001). Secretory expression of human growth hormone in *Saccharomyces cerevisiae* using three different leader sequences. Biotechnol. Bioprocess Eng. 6, 306-309.

Chang, C.M., Rey, M., Bochner, B., Heyneker, H., Gray, G. (1987). High-level secretion of human growth hormone by Escherichia coli. Gene 55, 189-195.
